# 

**Published:** 1832-07

**Authors:** 


### LONDON MEDICAL AND PHYSICAL JOURNAL.

EDITED BY

JOHN NORTH, Esq. F.L.S.

MEMBER OF THE ROYAL COLLEGE OF SURGEONS, AND OF THE MEDICAL AND CHIRURGICAL SOCIETY OF LONDON;

AND

GILBERT T. BURNETT, Esq.

F.L.S. M.B. R.I. R.C.S. &c.

PROFESSOR OF BOTANY IN KING'S COLLEGE, LONDON, AND TO THE MEDICO-BOTANICAL SOCIETY.

(VOL. LXVIII.)

NEW SERIES, VOL. XIII.

Et quoniam variant morbi, variabimus artes;

Mille mali species, mille salutis erunt.

45601

LONDON:

JOHN SOUTER, 73, ST. PAUL'S CHURCH-YARD;

AND TO BE HAD OF ALL MEDICAL BOOKSELLERS.

1832.

W1
LO 31

ADLARD, PRINTERS, BARTHOLOMEW CLOSE.

### MONTHLY LIST OF MEDICAL BOOKS.

[*Medical Works cannot be entered on this List unless a copy be sent for the purpose, the titles of Books having frequently been sent to us as published which have not been published for weeks, or even months, after.*]

Part VIII. The Principles and Practice of Obstetric Medicine, in a Series of Systematic Dissertations on Midwifery, and on the Diseases of Women and Children. Illustrated by numerous Plates. By DAVID D. DAVIS, M.D., M.R.S.L., Professor of Midwifery in the University of London, &c.—4to. John Taylor, London.

The Cyclopædia of Practical Medicine. Edited by JOHN FORBES, M.D., A. TWEEDIE, M.D., and JOHN CONOLLY, M.D. Part VI. (published in Monthly Parts.)—Royal 8vo. Sherwood, Gilbert, and Piper; and Baldwin and Cradock, London.

A Treatise on the Nature, Causes, and Treatment of Spasmodic Cholera. By C. F. FAVELL, M.D.—8vo. pp. 81. Groombridge, London.

A Lecture, introductory to a Course on Midwifery and Diseases of Females and Children, delivered at the Anglesey Lying-in Hospital. By G. T. HAYDEN, Member of the Royal College of Surgeons, Ireland; Surgeon to the Anglesey Lying-in Hospital, &c. Printed at the request of the Pupils.—Dublin.

Practical Observations on the Medicinal Virtues of the Beulah Saline Spa, Norwood; and on the Disorders in which it is most efficacious, especially those connected with the Derangements of the Digestive Organs. By ARCHIBALD MAXFIELD, Member of the Royal College of Surgeons in London, and formerly House-Surgeon to St. Bartholomew's Hospital.—8vo. pp. 38. Highley, London.

Observations on the General Principles, and on the particular Nature and Treatment of various Species of Inflammation. By J. H. JAMES, Surgeon to the Devon and Exeter Hospital, and Consulting Surgeon to the Exeter Dispensary.—8vo. pp. 565. Renshaw and Rush, London.

An Essay on the Epidemic Cholera: being an Inquiry into its new or Contagious Character, including Remarks on the Treatment; as likewise Tables of the average Rate of Disease and Mortality, recently occurring in London. By JOHN WEBSTER, M.D., Member of the Royal College of Physicians, London; Physician to St. George's and St. James's Dispensary; Lecturer on Materia Medica and Pharmacy, London.—12mo. pp. 220. Burgess and Hill, London.

Two Lectures on the Primary and Secondary Treatment of Burns. By HENRY EARLE, F.R.S., Surgeon Extraordinary to the King, &c.—Pp. 59, with Engravings. Longman and Co.

### APOTHECARIES' HALL.

* NAMES OF GENTLEMEN to whom the Court of Examiners have granted Certificates:

MAY 31.

William John Carson, of Galloway
Patrick Charles, Putney
William Cole, Devonport
Edward Farrar, Greenwich
Edward John Grafton, Alcester
George Houlton, Killingholme
Charles Malim, Lincoln's Inn fields
Edward Martin, Doncaster
William Jones Rowland, Anglesey
John Stonehouse Stephens, Manchester
John Woodforde.

JUNE 7.

Thomas Robinson Bowling, Hammersmith
Francis Charles Howard, Hingham, Norfolk
George Provost Heyward, Aylesbury
William Jones, Fairfield, Glocester
William Kirk, Hedon

JUNE 14.

Charles Robert Bree, York
Michael William Henry, London
Mark Watson Manby, Rudham
Richard Pechey, Isleham, Cambridge
Owen Roberts, Anglesea
David Robert Grant Walker, Plymouth
Robert Wood, Bury, Lincolnshire

JUNE 21.

George Henry Butler, London
Edward Joseph Welchpool, Welchpool
John Nottingham, Snaith
Richard Davis Pritchard, Horndean
James Selleek, Millbrook
Edward Wild, Stourport.

JUNE 28.

John Best, Brighton
Dennis Cronin, Killarney
Philip Henry Holland, Manchester
Thomas Seagrave, Warminster
John Shaw, Manchester
Charles Sweeney, Market street
Charles Wilson, Yoxford.

